# Exploring the optimal factor structure of mind-wandering: Associations with neuroticism

**DOI:** 10.1371/journal.pone.0311733

**Published:** 2024-12-11

**Authors:** Joseph Diehl, Nicolas Camacho, Moria Smoski

**Affiliations:** 1 Department of Psychology & Neuroscience, Duke University, Durham, North Carolina, United States of America; 2 Department of Psychiatry and Behavioral Sciences, Duke University Medical Center, Durham, North Carolina, United States of America; University of Porto Faculty of Psychology and Educational Sciences: Universidade do Porto Faculdade de Psicologia e de Ciencias da Educacao, PORTUGAL

## Abstract

Mind-wandering is an essential cognitive process in which people engage for 30–50% of their waking day and is highly associated with neuroticism. The current study identified the factor structure of retrospective self-report items related to mind-wandering and perseverative cognition content and explored these associations with neuroticism. In an adult community sample (*N* = 309), items from the NYC Cognition Questionnaire, the Penn State Worry Questionnaire Short Form, and the Rumination Responses Brooding Subscale were entered into factor analyses to test the optimal factor structure of these items. We employed a structural model to investigate associations of mind-wandering facets with neuroticism. A correlated three factor solution best fit the data (CFI = .94, TLI = .93, SRMR = .07, RMSEA = .07). Bifactor models failed to provide evidence for a general mind-wandering construct above and beyond variance explained by mind-wandering and perseverative cognition facets. The structural model revealed differential associations of each facet with neuroticism. A wandering mind is not always an unhappy mind. Whereas worry and rumination are associated with *higher* levels of neuroticism, mind-wandering has other components that relate to positively valenced cognition and *lower* neuroticism. Adaptive and maladaptive mind-wandering should be tested together in future studies of personality and psychopathology.

## Introduction

Mind-wandering, as a sub-type of task-unrelated thought, is considered a core cognitive function [1]. Mind-wandering may occur for upwards of half of our waking time [2, 3] and is affected by one’s personal concerns and executive control abilities [4, 5]. Mind-wandering is linked with notable benefits, including allowing us to connect our past and future selves together, helping us make successful long-term plans, supplying a forum for creative inspiration, and providing relief from tedious or monotonous situations [2, 6]. Despite evidence for its benefits, other studies show an association between mind-wandering and negative consequences such as deficits in reading comprehension, disruptions in working memory, car accidents, and problems with workplace functioning [7]. This mixed evidence indicates that mind-wandering is not inherently costly nor beneficial, and instead its impact depends on its content [2, 8].

The content of any given mind-wandering episode can span numerous categories including emotional valence, temporal orientation, intensity, self-reference, and internal vs. external orientation [2, 9]. Mind-wandering content has been examined via retrospective self-report, daily-life studies, and laboratory tasks. Recent work has begun to unpack the differential roles of mind-wandering content in laboratory settings [10, 11] and daily-life studies [12]. We seek to provide further clarity to retrospective self-reporting of mind-wandering content on the important dimensions of emotional valence and temporal orientation.

In addition to paying attention to mind-wandering content, recent work highlights the importance of considering automatic and deliberate constraints as dimensions of task-unrelated thought. To this end, the Dynamic Framework of Thought [13] differentiates more freely moving mind-wandering from sticky and repetitive types of task-unrelated thought, such as rumination (past-focused) and worry (future-focused), collectively known as Perseverative Negative Thinking (PNT). While mind-wandering and PNT are both task-unrelated, the mind may move freely from topic to topic in mind-wandering while getting stuck in the same topic repetitively in PNT [13]. Numerous studies implicate the role of PNT in the onset and maintenance of psychopathology [14].

Neuroticism is one of the Big Five personality traits [15], characterized by having a greater tendency to experience negative emotions. Studies have linked higher levels of neuroticism with poor mental and physical health [16] and increased risk for all-cause mortality [17]. Neuroticism has been shown to be a robust longitudinal predictor of the development and persistence of internalizing disorders, such as anxiety and depression, over a 10-year period [18]. Similarly, a meta-analysis revealed that neuroticism was strongly associated with a wide range of psychiatric disorders, including mood disorders, anxiety disorders, and substance use disorders [19]. A large-scale meta-analysis by Jokela and colleagues found that neuroticism was a robust predictor of increased mortality risk through cardiovascular diseases, cancer, and respiratory disorders [20]. Neuroticism has also been linked to gastrointestinal issues, headaches, and chronic pain [21].

Despite abundant evidence for the drawbacks of heightened neuroticism, a more mechanistic understanding of what may cause or be a product of neuroticism is still developing. When characterized only by its frequency, mind-wandering is consistently linked to neuroticism [22, 23]. A longitudinal study found baseline levels of mind-wandering predicted increases in neuroticism over a one-year period [24]. Smallwood and O’Connor found that individuals high in neuroticism reported more frequent and more negative thoughts during mind-wandering episodes, indicating that higher levels of neuroticism were associated with increased frequency and negative content of mind-wandering episodes [25]. Evidence suggests that PNT may account for links between neuroticism and internalizing symptoms [26]. Another study examined the associations of neuroticism and mind-wandering in laboratory and daily-life settings, finding that neuroticism predicted mind-wandering rate in laboratory tasks but not daily life [12]. These studies largely illustrate that task-unrelated thoughts and neuroticism are closely linked and may share a reciprocal relationship, influencing and predicting each other over time. However, evidence that considers how considerations of mind-wandering content and PNT may be associated with neuroticism in the same study remain unexplored.

While cited studies have defined neuroticism comprehensively, many of these studies have defined mind-wandering solely in terms of frequency without regard to content or simply conflated all types of task-unrelated thoughts under one umbrella term (most often as mind-wandering or PNT). In contrast, studies that consider different mind-wandering content have showed different associations between mind-wandering and affect. For example, subjects higher in neuroticism reported more racing thoughts and less clear thinking when their mind wandered [12]. In another study, negative mood was the only significant predictor of negatively-valenced mind-wandering [10]. In contrast, when mind-wandering was rated as personally interesting by participants, it was associated with improved mood [27]. Another study showed that self-reported negative affect was at its lowest when mind-wandering was rated by participants as positively valenced [28]. In line with the Dynamic Framework of Thought, Ottaviani et al. examined the separate contributions of PNT and less perseverative versions of mind-wandering [29]. In a 20-minute thought probing test, non-perseverative mind-wandering was not associated with physiological reactivity or low mood, but PNT (defined by the authors as a negatively-valenced, rigid, and inflexible type of task-unrelated thought) was significantly associated with less heart rate variability and lower mood [29]. These results merit further investigation into whether PNT is associated with neuroticism above and beyond other kinds of task-unrelated thinking.

As task-unrelated thought is heterogenous, it is important to clarify whether its association with neuroticism differs depending on the content of mind-wandering. The identification of distinct facets of task-unrelated thoughts will facilitate a better understanding of its adaptive and maladaptive aspects. In addition, different facets may be positively or negatively associated with neuroticism. One technique appropriate for investigating data-driven facets of a given construct is factor analysis, in which linear relations between observed variables are explained by unobserved, latent variables [30]. In studies for which we do not have prior evidence to hypothesize the factor structure with a high degree of certainty, exploratory factor analysis (EFA) is the most proper application of factor analysis as it does not constrain the factor structure in any way. As such, an investigation of commonly used retrospective self-report scales assessing the valence and temporal orientation of mind-wandering content and PNT in an EFA framework would be a useful contribution to the literature to enhance our understanding of the dimensionality of task-unrelated thoughts and whether they are differentially associated with neuroticism.

### The current study

The first aim of the current study is to empirically derive facets of mind-wandering in a series of factor analyses. Given the exploratory nature of the study, we had no a priori hypotheses, but expected PNT to be distinguished from other forms of mind-wandering based on prior findings [29]. To further investigate whether an overarching task-unrelated thought factor best represents the data as a hierarchical structure while also allowing for specific subscale factors to account for variance in the data, we employed two bifactor models. Given the frequency with which mind-wandering occurs, and the known relationship between PNT and neuroticism, understanding the factor structure of retrospective reports of task-unrelated thought content will help inform the role of task-unrelated thoughts in promoting or protecting against negative affectivity. Accordingly, the second aim of the current study was to investigate associations between the optimal measurement model of PNT and mind-wandering content with neuroticism within an SEM framework.

## Materials and methods

### Participants

Our sample was comprised of data from 309 adults enrolled in the NKI Enhanced Rockland Sample (eNKI-RS) [31]. As a community representative sample, the eNKI-RS has minimal exclusion criteria, and thus includes some participants meeting criteria for one or more DSM-IV-TR Axis 1 diagnoses. The mean age of the sample was 58.26 [*SD* = 9.61, range = 30–70]. For a full demographic breakdown, see Table 1.

**Table 1:** Demographics.

	*N*	%
Gender		
Male	94	30.4
Female	215	69.6
Household Income per Year		
25,000 or less	26	8.4
25,000–50,000	27	8.7
50,000–100,000	80	25.8
100,000–200,000	98	31.7
200,000+	39	12.7
Did Not Disclose	39	12.7
Marital status		
Married/living together	206	66.7
Divorced/separated	56	18.1
Widowed	14	4.5
Never married	33	10.7
Ethnicity		
White	252	81.6
African American/Black	18	5.8
Hispanic/Latinx	23	7.4
Asian	8	2.6
Other	8	2.6

### The NYC-Cognition Questionnaire (NYCQ)

The New York Cognition Questionnaire (NYC-Q) is a self-report tool that assesses mind-wandering by querying different types of thoughts and feelings experienced while performing a particular activity [32]. It consists of two sections, the first containing 23 questions about the content of thoughts, the second containing eight questions about the form that these thoughts take. We used the first section only, as we were primarily interested in the content of mind-wandering. For each question, subjects were asked to indicate how well each statement described their thoughts on a scale from 1 [‘‘Completely did not describe my thoughts”] to 9 [‘‘Completely did describe my thoughts.”] One example statement is, “I thought about an interaction with somebody that took place in the past.” All subjects completed the questionnaire at the end of a resting state fMRI scanning session. Participants were neither encouraged nor discouraged to let their minds wander. The original scale identified five different facets of mind-wandering: past, positive, future, negative, friends [32]. As the scale is in the public domain, a full copy of the scale with item descriptions is included Table 2. Internal consistency for this sample was excellent [α = .93].

**Table 2:** Standardized factor loadings for final three-factor EFA.

Item	Description	NP	PFI	PNT
NYCQ_01	I thought about things I am currently worried about	0.585*	0.227*	0.160*
NYCQ_02	I thought about people I have just recently met	0.505*	0.260*	0.055
NYCQ_03	I thought of people I have known for a long time (friends)	0.488*	0.429*	0.014
NYCQ_04	I thought about members of my family	0.296*	0.567*	0.017
NYCQ_05	I thought about an event that took place earlier today	0.378*	0.334*	0.041
NYCQ_06	I thought about an interaction I may possibly have in the future	0.350*	0.595*	-0.088
NYCQ_07	I thought about an interaction with somebody that took place in the past	0.573*	0.421*	-0.084
NYCQ_09	I thought about something that made me feel guilty	0.849*	-0.094	-0.058
NYCQ_10	I thought about an event that may take place later today	-0.053	0.775*	0.135*
NYCQ_12	I thought about something that happened a long time ago in the past	0.638*	0.215*	-0.073
NYCQ_13	I thought about something that made me angry	0.874*	-0.059	0.067
NYCQ_14	I thought about something that made me happy	0.073	0.793*	-0.079
NYCQ_16	I thought about something that made me calm	-0.184	0.798*	0.080*
NYCQ_17	I thought about something that made me sad	0.780*	-0.014	0.120*
NYCQ_18	I thought about something that is important to me	0.126*	0.771*	-0.017
NYCQ_19	I thought about something that could still happen today	0.026	0.762*	0.131*
NYCQ_20	I thought about something that may take place in the distant future	0.324*	0.543*	-0.034
NYCQ_21	I thought about something that could take place in the near future (days or weeks but not today)	0.122	0.754*	-0.036
NYCQ_22	I thought about personal worries	0.687*	0.142*	0.274*
NYCQ_23	I thought about something that happened in a place far away from where I am now	0.513*	0.268*	-0.027
PSWQ_01	If I do not have enough time to do everything, I do not worry about it.	-0.114	0.057	0.411*
PSWQ_04	Many situations make me worry.	0.141*	-0.058	0.815*
PSWQ_05	I know I should not worry about things, but I just cannot help it.	0.001	0.035	0.896
PSWQ_06	When I am under pressure I worry a lot.	0.062	0.055	0.762*
PSWQ_07	I am always worrying about something.	0.017	0.043	0.864*
PSWQ_09	As soon as I finish one task, I start to worry about everything else I have to do.	0.048	0.086	0.746*
PSWQ_12	I have been a worrier all my life.	-0.039	0.077	0.803*
PSWQ_13	I notice that I have been worrying about things.	0.123*	-0.004	0.794*
RRS_005	Think "What am I doing to deserve this?"	0.232*	-0.139	0.516*
RRS_010	Think "Why do I always react this way?"	-0.034	0.069	0.747*
RRS_013	Think about a recent situation, wishing it had gone better	0.138	0.001	0.545*
RRS_015	Think "Why do I have problems other people don’t have"	0.222*	0.012	0.613*
RRS_016	Think "Why can’t I handle things better?"	0.109	0.005	0.772*

*p < .05.

### Ruminative Response Scale-Short form (RRS-S)

The RRS-SF is one of the most widely used self-reported measures of trait rumination, comprising ten items, and describing the factors of reflection and brooding [33]. Studies have supported a reflection—brooding two-factor model and the scale has been validated in clinical and non-clinical populations [34, 35]. We used the 5-item brooding subscale as this most closely resembles the clinically maladaptive aspects of rumination [36], with scores on 4-point scale ranging from 1 (almost never) to 4 (almost always). The mean score for this sample was 8.11 (*SD* = 2.65, range = 5–20), which was slightly lower than one other general population sample [37]. However, total RRS-SF [including the reflection subscale] scores were in line with other total RRS-SF scores [38, 39]. Internal consistency for this sample was good [α = .82]. The items from this scale are reprinted in Table 2 with permission (CCC license number 5402531141033).

### Penn State Worry Questionnaire Short-Form (PSWQ-SF)

The PSWQ-SF is an 8-item measure of trait worry [40]. The scale was initially designed to address poor model fit of the full version with older adult samples. The PSWQ-SF demonstrates desirable psychometric properties when tested against the full scale [41]. The PSWQ-SF is a 5-point scale from 1 (not at all typical of me) to 5 (very typical of me). The mean score for this sample was 20.09 (*SD* = 6.69, range = 8–39), which was comparable to the means of three other general population samples [42]. Internal consistency for this sample was good (α = .84). The items from this scale are reprinted in Table 2 with permission (CCC license number 5356550147415).

### NEO Five Factor Inventory (NEO-FFI-3)

The NEO-FFI-3 is a 60-item psychological personality inventory that assesses based on the five-factor model: Openness to Experience, Conscientiousness, Extraversion, Agreeableness, and Neuroticism. Participants are asked to select the response that best represents their opinion on a 5-point scale from 0 (strongly agree) to 4 (strongly disagree). Given our research goals, we only used data from neuroticism, which was represented by 12 items. Internal consistency for the sample was good (α = .88).

### Structured Clinical Interview for DSM-IV-TR Axis I disorders—Non-Patient edition [SCID-I/NP]

The SCID-I/NP is a diagnostic semi-structured interview designed to assess current and past episodes of psychopathology in adults, according to DSM-IV criteria [43]. Probes and objective criteria are provided to rate individual symptoms. Domains assessed include anxiety (including obsessive-compulsive and related disorders), depression and mood, eating behavior, psychotic behavior, substance use, and trauma and stressor-related disorders. In this sample 36.5% of participants met criteria for at least one current DSM-IV disorder and 42.7% met criteria for at least one past DSM-IV disorder. For specific rates of diagnoses and diagnostic categories, see S2 Table.

### Ethics statement and procedure

Institutional Review Board (IRB) approval was obtained for the original development of the dataset at the Nathan Kline Institute (Phase I #226781 and Phase II #239708). Written informed consent was obtained for all study participants. Procedures for the data collection are described in the original study [31]. Demographic factors, PSWQ and RRS measures were collected via self-report. Diagnostic categories were assessed via SCID-I/NP interviews. Participants completed the NYC-Q following a 10-minute resting state fMRI scan during which they were instructed that they should keep their eyes on a fixation cross. The present study was determined to be IRB exempt by the Duke Health IRB (Protocol ID: Pro00107386). Data from the sample for this project were accessed between January 5 and April 27, 2022. The authors did not have access to information that could identify individual participants.

### Data preparation

We report how we determined our sample size, all data exclusions, all manipulations, and all measures in the study. No multivariate outliers were found using Mahalanobis Distance or Cook’s Distance. We ran bivariate correlations of the NYC-Q, RRS-SF, and PSWQ-SF items to check for multicollinearity and eliminated NYC-Q item 15 (“I thought of something that made me cheerful”) from the analysis because of its correlation of.91 with NYC-Q item 14 (“I thought of something that made me happy”). Distribution plots of individual items of the NYC-Q, RRS-SF, and PSWQ-SF revealed positive skews in many items. As a result, we truncated the 9-point NYC-Q items into 5-point items, where 1 remained its own bin, and we merged the rest into combined bins([i.e., 2 with 3, 4 with 5, 6 with 7, and 8 with 9) following previous recommendations [36, 44]. The NYC-Q thus became an ordered categorical scale with scores ranging from 1 to 5, resembling the ordered categorical nature of the RRS-SF and PSWQ-SF scales (see S1 Table for a correlation matrix of all items). We next tested for multivariate normality using Mardia’s test, which is based on multivariate extensions of skewness and kurtosis [45]. As expected, given the distribution of the data, this test strongly rejected the null hypothesis in favor of non-normality. Next, we assessed for sampling adequacy to make sure an exploratory factor analysis (EFA) was possible with our data. Given the lack of multivariate normality in our data, we used Kaiser-Meyer-Olkin Test [46]. The overall measure of sampling adequacy (MSA) was 0.89 and all individual MSA values were above 0.8, indicating suitability of the data for factor analysis. We considered three tests to identify the optimal factor solution of our scales, including 1) principal axes and 2) principal component parallel analyses [47] and 3) visual inspection of the eigenvalue distribution from the resulting scree plot using the commonly used Kaiser-Guttman criterion (eigenvalues > 1). Principal axes and principal components parallel analyses both suggested the retention of three factors. The application of the Kaiser-Guttman criterion suggested a retention of six factors; thus, we investigated factor solutions ranging from three to six.

### Analyses

#### Identification of optimal mind-wandering structure

**Fig 1:**
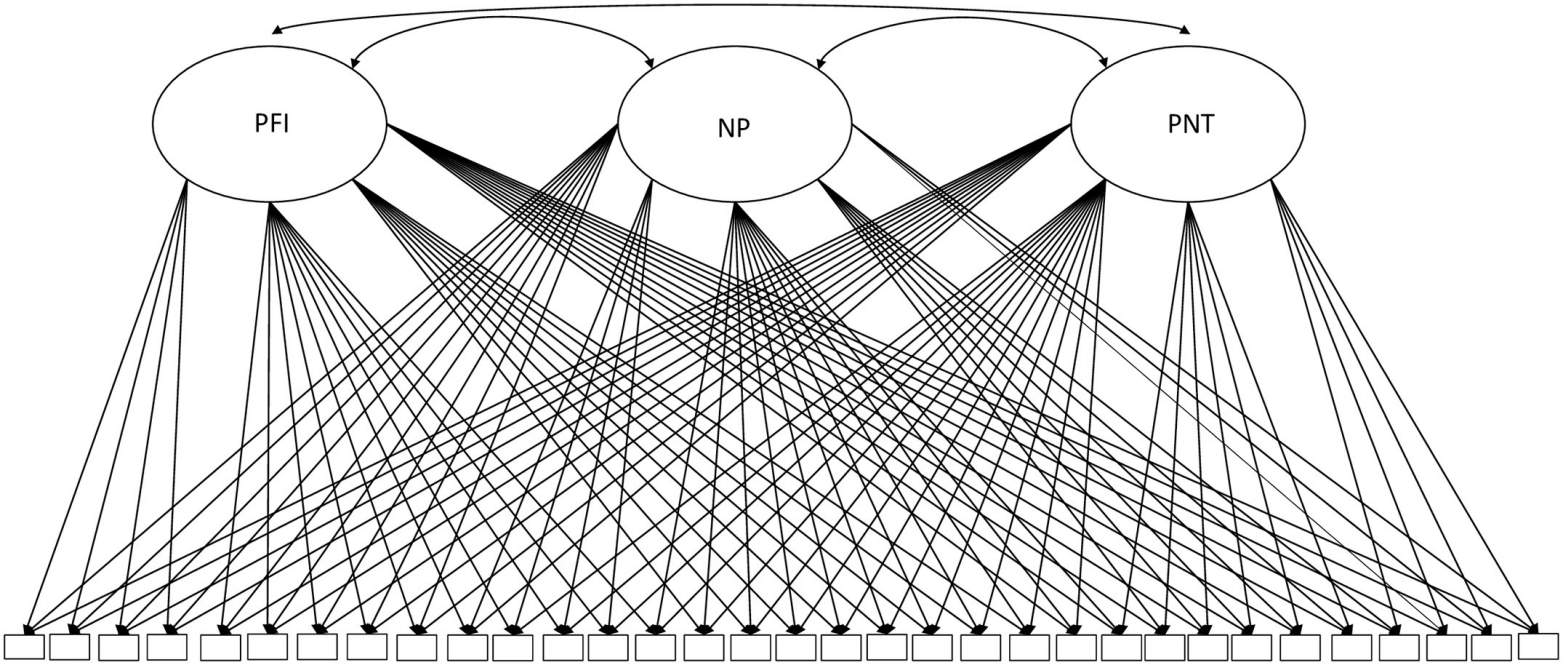
Conceptual model of EFA of mind-wandering.

Scripts for analyses are available at https://osf.io/87cyw/. We conducted all EFAs and CFAs using Mplus Version 8.6. Given that all our scales were 4- and 5-point scales, we treated each scale as ordered categorical. At each factor level, we evaluated fit based on values of the comparative fit index (CFI) [48], the Tucker-Lewis Index (TLI) [49], the standardized root mean square residual (SRMR) [50], and the root mean square of approximation [51]. Bentler and Bonett suggested that models with indices such as CFI/TLI ≥ 0.90, RMSEA ≤.08, and SRMR ≤.08 were acceptable [52]; other studies support interpretational benchmarks for good fit of CFI and TLI ≥ 0.95 and RMSEA ≤ 0.06 [50]. Adjudication of optimal model fit was conducted by consulting the contents of the items, and the fit and parsimony of a given model. That is, for models with identical fit, we deferred to the more parsimonious model as ideal. We used the oblique Crawford-Ferguson varimax rotation for the EFA and WLSMV estimator based on a polychoric matrix of the data due to its robustness with categorical and skewed data [53]. To assess the extent to which results were dependent on the selection of estimation method, we subsequently ran the EFA with a maximum likelihood estimation method robust to non-normality (MLR). For a conceptual model of this analysis, refer to Fig 1.

#### Bifactor models

We next employed a bifactor model under a confirmatory approach to separate the general effect shared by the items onto which the mind-wandering facets loaded and the specific effects associated with each mind-wandering facet (see conceptual model in S1 Fig). We assumed the relations among the general and specific facets to be orthogonal in line with the classical bifactor approach and hypothesized that the specific facets would account for variance over and above the general mind-wandering factor. We also used this model to identify whether any mind-wandering facet would no longer remain a unique contributor, after taking into account the common variance among the full set of items [54]. In other words, we tested for the idea that variance in a specific mind-wandering facet (e.g., mind-wandering1) could be entirely redundant with that of general mind-wandering, whereas other mind-wandering facets (e.g., mind-wandering2, mind-wandering3) remained as specific factors, even after partialling out general mind-wandering. In this scenario, mind-wandering1 would no longer be a specific factor in the final bifactor model. In addition to using our classic bifactor approach we also used bifactor exploratory structural equation modeling (ESEM) to estimate the model. The ESEM approach combines feature of confirmatory and exploratory factor analysis by allowing off-loadings to be estimated freely. We employed this analysis using a target rotation, which specifies key construct indicators (as in CFA) with all cross‐loadings being freely estimated (as in EFA) but “targeted” to be as close to zero as possible without constraining those cross-loadings to zero (as in CFA) [55].

For additional interpretations for the bifactor models, we employed multiple descriptive indices including values of explained common variance, omega hierarchical (ωh), omega hierarchical subscale (ωhs), and coefficient H [56]. ECV scores sum to 1.0 and divide common variance as a function of our modeled latent variables. If mind-wandering is unidimensional, we would expect to see ECV values of.70 or higher for our mind-wandering factor. Ωh indexes the proportion of systematic variance in a unit-weighted composite of all items attributable to the general factor. If mind-wandering is unidimensional, we would expect to see an ωh score of greater than.75 [49]. Ωhs is the comparable proportion for each specific factor after variance attributable to the general factor is accounted for. Values less than.50 for any subfactor would suggest caution in interpreting scores as indicating content beyond that attributable to a general TUT factor [57]. Coefficient H is a measure of construct reliability that reflects the correlation between a factor and a composite of items [58]. Values of.80 would indicate well-defined latent variables likely to replicate across studies, while scores of less than.70 for a given factor would raise concern about the use of their items to assess the specific domain they are supposed to reflect as opposed to general mind-wandering.

#### Structural model—Associations with neuroticism

Using our determined measurement model of mind-wandering, we investigated associations of our EFA-derived facets of mind-wandering with neuroticism within an SEM framework. All scales were treated as ordered categorical and we used WLSMV estimation. Fit was evaluated as specified above, evaluating values of CFI, TLI, RMSEA, and SRMR.

## Results

### Measurement models

Based on visual inspection of scree plot and eigenvalue analyses, factor loadings for 3–6 factors are included in S3–S6 Tables. The three-factor solution estimated cleanly and fit the data acceptably (CFI = .94, TLI = .93, SRMR = .06, RMSEA = .07). Note that our specific facets are written in italics for clarity. The first factor of the three-factor solution contained mind-wandering items with state-based negative and/or past-oriented content and has been named “*Negative/Past*.” The second factor contained primarily positive, future, and interaction-related content, and was named “*Positive/Future*.” The third factor contained the items from the PSWQ-SF and RRS-SF, consistent with past studies of perseverative negative thinking, and has been named “*PNT*.” *Negative/Past* and *Positive/Future* correlated significantly (*r* = .50, *p* < .001), *Negative/Past* and *PNT* correlated significantly (*r* = .25, *p* < .001), and *PNT* and *Positive/Future* correlated significantly (*r* = .12, *p* < .001). While a 4-factor model also estimated cleanly and fit the data well (CFI = .96, TLI = .95, SRMR = .05, RMSEA = .06), the fourth factor only yielded two statistically significant and unique indicators—thus it was deemed uninterpretable (S4 Table). Similarly, the 5- and 6- factor solutions estimated cleanly and yielded increasingly better fits to the data (5-factor: CFI = .97, TLI = .96, SRMR = .04, RMSEA = .05; 6-factor: CFI = .98, TLI = .97, SRMR = .04, RMSEA = .05), yet did not provide unique and statistically significant factor indicators for certain factors (S5 and S6 Tables). Thus, the three-factor solution provided the best balance of fit, parsimony, and interpretability (S3 Table).

A closer look at the loading pattern of the three factor solution led to the removal of two NYC-Q items (“I thought about something that happened at a place very close to me;” “I thought about something that happened in the recent past (last couple of days but not today)”) due to cross-loadings and lack of theoretical clarity, which yielded near-identical fit indices to the previous three-factor solution (CFI = .94, TLI = .93, SRMR = .06, RMSEA = .07; Table 2). Similar to the previous analysis, we found significant correlations between *Positive/Future* and *Negative/Past* (*r* = .46, *p* < .001), *PNT* and *Negative/Past* (*r* = .26, *p* < .001), as well as between *Positive/Future* and *PNT* (*r* = .13, *p* < .001. In the sensitivity analysis, we tested the three-factor solution using the same rotation but with the MLR estimator, which is also robust to categorical and skewed data but does not permit traditional fit indices. This analysis solution yielded a similar factor solution as with the WLSMV estimator, wherein the same factors of *Negative/Past*, *Positive/Future*, and *PNT* emerged (AIC = 23897.07, BIC = 24783.334, *N-*adjusted BIC = 24031.72). For information regarding specific factor loadings, see S7 Table.

To simultaneously test the relative contribution of the general mind-wandering factor and our EFA-guided specific factors, we next employed a CFA bifactor model. The three-factor bifactor CFA solution estimated cleanly and fit the data acceptably (CFI = .95, TLI = .94, SRMR = .07, RMSEA = .06). Because the general factor did not significantly load onto all items and the specific factors loaded significantly onto their respective items, mind-wandering was not considered to be a dominant general factor. Data for every item loading can be found in S8 Table. Beyond item loadings we used additional unidimensionality tests detailed in our methods. The ECV of the general factor was.52, inconsistent with a unidimensional mind-wandering structure. ECVs for the *Negative/Past*, *Positive/Future*, and PNT factors were.13,.41 and.85, respectively. Ωh for the general mind-wandering factor was.73 while ωhs values for *Negative/Past*, *Positive/Future*, and *PNT* were.03,.37, and.77, respectively. Reliability as indexed by ω was.96. Altogether, these indices do not support the existence of a unidimensional mind-wandering factor (though our value of.96 for coefficient H for a single mind-wandering factor indicates a well-defined, replicable source of common variance).

The bifactor exploratory structural equation modeling model fit the data well, producing similar fit indices as the bifactor model (CFI = .96, TLI = .95, SRMR = .05, RMSEA = .06). Loadings on the general factor were mostly high and significant; loadings on the specific factors were similar to the bifactor model (see S9 Table for further information on all factor loadings). ECV was.47 for the general factor and.13,.08, and.31 for *Negative/Past*, *Positive/Future*, and *PNT*, respectively. The value of ωh was.75 and values of ωhs were.04,.03, and.13 for *Negative/Past*, *Positive/Future*, and *PNT*, respectively. The value of coefficient H was.95 and reliability as indexed by ω was.96. Similar to the bifactor model, these factor loadings and additional indices do not support the existence of a unidimensional mind-wandering factor.

### Structural model

**Fig 2:**
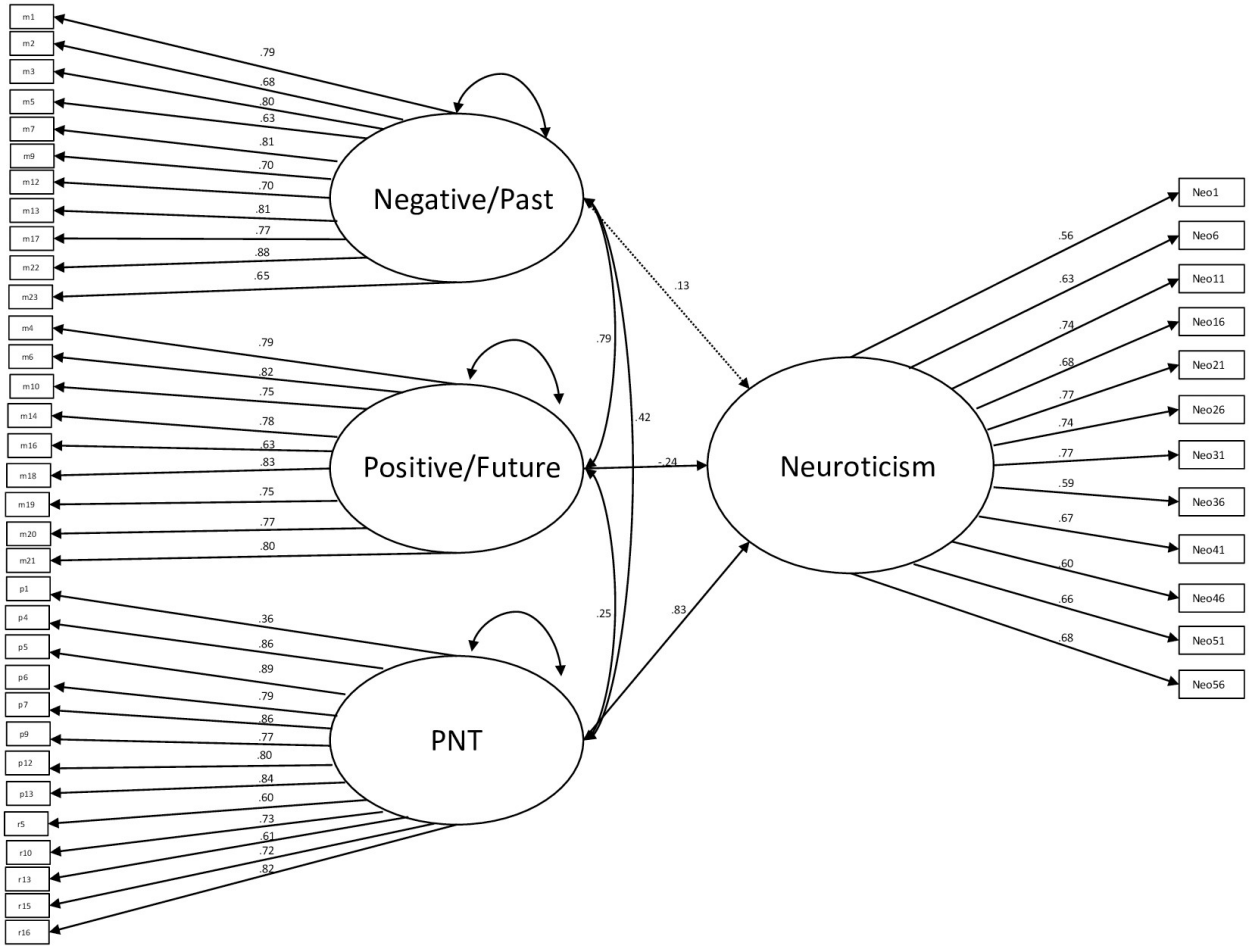
Structural model of associations between mind-wandering facets and neuroticism.

Based on the various measurement model procedures, it was determined that a correlated 3-factor model of mind-wandering with the facets of *Negative/Past*, *Positive/Future*, and *PNT* best represented the data. Employing a CFA approach using this measurement model and regressing each facet to a latent neuroticism factor (italicized for clarity as *neuroticism*) yielded an acceptable fit to the data (CFI = .94, TLI = .93, SRMR = .07, RMSEA = .05). All items loaded significantly onto their latent factors. A significant positive pathway emerged between *PNT* and *neuroticism* (0.83, *p* < .001), while a significant negative pathway emerged between *Positive/Future* and *neuroticism* (-0.22, *p* = .001). There was no significant pathway between *Negative/Past* and *neuroticism* (0.11, *p* = .15). Full results of this model can be viewed in Fig 2. We ran a sensitivity analysis this structural model taking out one of our rumination items and two of our neuroticism items given their qualitative similarity and high correlations. This model also provided acceptable fit of the data (CFI = .94, TLI = .94, SRMR = .07, RMSEA = .06) and the strength and significance of the model did not significantly change (see S2 Fig). We also present multiple regression results of associations of the same facets with the other personality variables covered in the NEO FFI in S10–S14 Tables (Extraversion, Openness, Agreeableness, Conscientiousness).

## Discussion

The current study examined the optimal factor structure of items describing the frequency and content of retrospective, self-reported mind-wandering and PNT, as measured by the NYC-Q, RRS-SF, and PSWQ-SF, in a community sample. We first employed an EFA of three- to six-factor solutions. A three-factor solution provided the optimal balance of fit and parsimony to our data. Guided by the results from our EFA, we next implemented a series of CFA approaches to further identify an optimal measurement model for mind-wandering. We tested three bifactor models, which all estimated cleanly and provided acceptable fit to the data. However, further investigation identified that these data were not best conceptualized by a general mind-wandering factor. These data were better defined by three correlated sub-factors of *Positive/Future*; *Negative/Past*; and *PNT*. Overall, these results suggest that PNT provides valuable additional context to the concept of mind-wandering, and that correlated latent constructs of *Positive/Future*; *Negative/Past*; and *PNT* should be tested together in future studies. Consistent with our findings, a recent experience sampling study with adolescents demonstrated that negatively valenced, past‐oriented and self‐focused thoughts were each independently associated with higher concurrent negative affect above and beyond general mind-wandering frequency [59]. Thus, generalizing only one of these factors as having implications for overall mind-wandering may be misguided.

Next, we employed a structural model, in which our three mind-wandering facets were regressed onto a latent neuroticism factor. In this model, the pathway between *Negative/Past* and *neuroticism* was insignificant, the pathway between *Positive/Future* and *neuroticism* was significant in a negative direction, and the pathway between *PNT* and *neuroticism* was significant in a positive direction. These results indicate that *Positive/Future* styles of mind-wandering may be contrary to trait neuroticism. However, consistent with prior literature, PNT is associated with trait neuroticism [60]. Results highlight that task-unrelated negative thoughts on their own are not associated with neuroticism, but only negative thoughts that are repetitive and long-lasting.

### The exploratory factor structure of mind-wandering

Even though mind-wandering is something we engage in for a significant portion of time daily, the extant literature on task-unrelated thoughts as a construct is largely inconsistent. Studies routinely consider mind-wandering in a unidimensional context. The terms “mind-wandering” and “perseverative negative thinking” are often used interchangeably. Results from our EFA indicate that mind-wandering is best described by distinct facets of *PNT*, *Positive/Future*, and *Negative/Past* are all distinct facets of task-unrelated thought. These results also support a difference between temporary negative mind-wandering (the *Negative/Past* factor) and a more general tendency to perseverate on negative content (i.e., *PNT*). The higher correlation between *Positive/Future* and *Negative/Past* than between *Negative/Past* and *PNT* suggests when mind-wandering is not perseverative, people may switch between different types of mind-wandering (both positively and negatively valenced) with more frequency. Given the dearth of studies that examine transient state mind-wandering and PNT in tandem, the results of our EFA establish a precedence for understanding the respective contributions to adaptive and maladaptive mind-wandering to a general construct of mind-wandering.

There is extant literature in support of our correlated factor structure of *Positive/Future*, *Negative/Past*, and *PNT*. For example, mind-wandering focused on the future was associated with a subsequent more positive mood, indicating that positive and future-oriented mind-wandering may cluster together [61]. Conversely, past-focused mind-wandering was associated with reports of negative mood, indicating past and negative-mind-wandering may cluster together [25, 61]. However, these temporal associations may not apply in PNT, wherein both past- (rumination) and future-focused (worry) constructs both provide meaningful contribution to the latent PNT construct. Temporary negative and past-oriented mind-wandering may be distinguished from PNT by degrees of automatic constraint [13] and cognitive flexibility [29]. In other words, it may be useful at times to think about one’s mistakes in the past or express some concern for the future if it helps one successfully plan and work toward future goals, but the inability to extricate oneself from the negatively-valenced content of such thoughts becomes harmful. Working memory capacity may be another distinguishing variable. One recent study found that higher working memory ability was positively associated with within-person and within-task variability of task-unrelated thoughts, suggesting that subjects with better cognitive control may have mind-wandered for less time during each instance, and shifted their mental context back to the ongoing task more frequently than did subjects with poorer control [11]. In sum, evidence from this study that *PNT*, *Negative Past*, and *Positive/Future* are significantly correlated with each other (even after accounting for common variance) provides support for examining all three in tandem. Furthermore, these results raise the question if propensity to mind-wandering may provide some degree of risk for engaging in PNT, even if the constructs of *Negative/Past* and *Positive/Future* may not be as harmful in and of themselves. These results are preliminary, as research is just beginning to disentangle these effects and additional dimensional assessments of the phenomenology of mind-wandering are warranted [24].

Regarding the NYC-Q, results from our EFA provide support for a more parsimonious model, wherein two factors (*Positive/Future*, *Negative/Past*) sufficiently capture the variance of items from this scale when introducing PNT-related items. As discussed in the prior paragraph, there is theoretical support for each of our non-perseverative mind-wandering factors. Results from a previously published factor analysis of the NYC-Q provided support for a five-factor solution [32]. However, some of the questions used for factor interpretation in their analysis were significantly cross-loaded (e.g., NYCQ-2, NYCQ-3, NYCQ-9, NYCQ-13, NYCQ-18, NYCQ-19), calling into question the interpretability of the five-factor model. In future studies using the NYC-Q, we encourage future attempts to replicate its factor structure testing both a five and two-factor solution.

### Additional measurement models of mind-wandering

While each of the facets were significantly related, we aimed to examine whether the data supported their subsumption under a general task-unrelated thought factor (i.e., testing whether mind-wandering is unidimensional). To simultaneously test the relative contributions of the general mind-wandering factor and our EFA-guided specific factors, we employed two bifactor models and, beyond factor interpretation, employed multiple descriptive indices (ECV, Omega, etc.) Despite good fit to the data, in both models, results failed to confirm the existence of an overarching mind-wandering factor. To our knowledge, no prior study has tested evidence for a general mind-wandering factor that considers perseverative negative cognition alongside positively-valenced thoughts. Equivocal results from both bifactor models provide support for the superiority for a correlated factors model (*Positive/Future*, *Negative/Past*, and *PNT*). Thus, in future studies, it is recommended to use correlated latent factors of mind-wandering rather than a single, latent construct. These results provide further support for the notion that mind-wandering has various aspects of functioning, and it is not a uniformly maladaptive construct. These results are subject to further replication and future studies should test both bifactor and correlated factor models of self-report mind-wandering.

### Structural model—Associations with neuroticism

A more nuanced interpretation of mind-wandering is warranted in terms of associations with mood and psychopathology instead of the popular narrative that a wandering mind is an unhappy mind [3]. When defined as a unidimensional construct, mind-wandering was significantly associated with higher internalizing problems, externalizing problems, executive functioning deficits, thought disorder problems, somatic complaints, and emotion dysregulation in one clinical sample [62]. However, our results suggest associations may not be as straightforward as previously reported. Specifically, *Negative/Past* showed no significant association with *neuroticism*, while *Positive/Future* and *PNT* showed significant associations with *neuroticism* in opposing directions. Given other mixed associations between mind-wandering and neuroticism [10, 60, 63, 64], parsing apart mind-wandering by content in addition to frequency is crucial to better understand how different styles of mind-wandering and related to negative emotionality.

Several studies have supported associations between positively-oriented types of mind-wandering and enhanced positive mood or reduced negative mood. For example, when participants mind wandered to a topic they were inherently highly interested in, they reported improved mood [27]. In another study of university students, self-reported negative affect was at its lowest when mind-wandering was rated by participants as positively valenced [28]. This effect was not moderated by self-reported rumination, suggesting standalone utility in examining positively valenced mind-wandering alongside other forms of mind-wandering. The rise in popularity of mindfulness, often defined by its qualities of nonjudgmental present-moment awareness, has helped to create a perception of task-focused thought as the best suited antidote to negative mood associated with mind-wandering, but these results suggest positively valenced, future-oriented, and interaction-oriented mind-wandering may also confer reduced risk for the propensity to experience negatively valenced mood. Future studies should test positively valenced, future-oriented, and interaction-oriented mind-wandering alongside mindfulness as solutions for reducing mind-wandering-oriented psychopathology. Perhaps the two could also be combined—individuals could be encouraged to leverage mindfulness skills to shift the content of their mind-wandering experiences towards pleasant topics.

Our results of a significant pathway between PNT and neuroticism are widely supported by the literature [60]. In fact, PNT is speculated to be a mechanistic maintainer in the association between neuroticism and psychopathology [64]. Significantly, even when allowing PNT to covary with other types of mind-wandering, the pathway between PNT and neuroticism remained strong and significant in our results (.83, *p* < .001), thus further confirming the utility of examining PNT as a key component of neuroticism. Given that neuroticism can be a modifiable target of treatment by interventions such as the Unified Protocol [65], future studies should explicitly investigate the reduction of PNT as a mechanism for neuroticism reduction. We note, however, that associations between neuroticism and mind-wandering content can vary between laboratory tasks and daily-life studies [e.g., 12]. Future studies should examine the extent to which retrospective self-reports of mind-wandering and PNT corroborate these other methods of assessing for mind-wandering.

Our results distinguish *PNT* from *Negative/Past* both in terms of contributions to a latent construct of mind-wandering and by associations with neuroticism. *Negative/Past* and *PNT* are likely distinguished by the degree to which negatively-valenced thoughts are perseverative. Studies have examined this distinction at the physiological level. Ottaviani tested 73 subjects using a task-based, heart rate variability (HRV) study [29]. Subjects who were simply wondering about their day or about their future tasks and responsibilities had higher HRV, and subjects who were ruminating and worrying had lower HRV. As higher HRV is an indication of more parasympathetic nervous system activity and is associated with adaptive emotion regulation, more transient mind-wandering about the future or with a positive valence may relate to emotional wellbeing while PNT may indicate the opposite.

While people may drift task-unrelated into negatively valenced and past oriented mind-wandering, our results posit that, as long as these thoughts are not perseverative, they likely do not contribute to increased risk for neuroticism. Furthermore, the higher correlation between *Positive/Future* and *Negative/Past* (than either with PNT) may suggest that people bounce back and forth with less maladaptive versions of mind-wandering more readily, while they have more difficulty extricating themselves from rumination or worry [66]. One explanatory process for these results suggests that *Positive/Future* and *Negative/Past* may be distinguished from *PNT* by the degree to which the thinking is guided. In *PNT*, people may feel drawn back to their thoughts while the unguided nature of *Positive/Future* and *Negative/Past* do not create the same attachment to this kind of cognition [67]. Supporting this theory, one experience sampling study identified freely moving thought as positively associated with a more positive affective state [67]. The fact that higher working memory ability has recently been associated with within-person and within-task variability of task-unrelated thoughts is another example that mind-wandering may not be inherently maladaptive if one does not get stuck in the same perseverative mental loop [11].

### Limitations and future directions

Due to the cross-sectional design of the study, we are unable to make any causal claims among our variables of interest. The sample was predominantly female, though sensitivity analyses did not identify significant differences between females and males on any construct scores except for PSWQ scores (S10 Table). Though the full sample was designed to be representative of the entire United States, the data available for the present study underrepresented Black, Indigenous, Latinx, Asian, and other minoritized samples at a national level. We encourage future replication of the factor structure of mind-wandering in more nationally-representative populations. The data presented in this study is retrospective self-report, which may be prone to bias and is only one way of assessing mind-wandering. While a multi-dimensional, exploratory study has been done with features of the phenomenology of mind-wandering [24], future studies should also include items based on PNT in these analyses. It should be noted that associations of these various facets of mind-wandering with neuroticism may depend on the sample. For example, in a hypothetical sample of patients diagnosed with social anxiety disorder, thoughts about future interactions with others, while associated with positive valence in our sample, may be more associated with negative valence given the phenomenological nature of social anxiety disorder. Future studies should endeavor to replicate our factor structure of mind-wandering and examine its associations with neuroticism in clinical samples. In requested supplementary analyses, we found significant associations between our different task-unrelated thought facets and other personality constructs, signifying neuroticism is not the only personality trait predicted by mind-wandering. There are other contexts of mind-wandering we did not consider such as the deliberate versus spontaneous nature of mind-wandering [e.g. 68, 69]. Vanucci & Chiorri [70] found that spontaneous mind-wandering was significantly related to rumination and not to reflection, while deliberate mind-wandering was significantly related to reflection but not rumination; thus, rumination may be experienced as much less deliberate than other more adaptive types of mind-wandering. According to the Dynamic Framework of Thought, mind-wandering is distinguished from PNT by its low deliberate and automatic constraints (contrasted with the constrained nature of rumination and worry).

Taken together, these results advocate for the inclusion of adaptive forms of mind-wandering together with perseverative cognition in studies that aim to investigate mind-wandering in both clinical and nonclinical samples.

## Supporting information

S1 Table Polychoric matrix of all items. (CSV)

S2 Table Diagnoses of sample. (XLSX)

S3 Table Standardized factor loadings, factor correlations, and fit indices for three-factor solution before MRIQ_8 and MRIQ_11 removal. (XLSX)

S4 Table Standardized factor loadings, factor correlations, and fit indices for four-factor EFA. (XLSX)

S5 Table Standardized factor loadings, factor correlations, and fit indices for five-factor EFA. (XLSX)

S6 Table Standardized factor loadings, factor correlations, and fit indices for six-factor EFA. (XLSX)

S7 Table Standardized factor loadings using MLR for three-factor model. (XLSX)

S8 Table Standardized factor loadings and fit indices for bifactor model. (XLSX)

S9 Table Standardized factor loadings and fit indices for bifactor ESEM model. (XLSX)

S10 Table Levine’s test for equality of variances between genders—Equal variances assumed. (XLSX)

S11 Table Multiple regression of mind-wandering facets with Extraversion. (XLSX)

S12 Table Multiple regression of mind-wandering facets with Openness. (XLSX)

S13 Table Linear regression of mind-wandering facets with Agreeableness. (XLSX)

S14 Table Linear regression of mind-wandering facets with Conscientiousness. (XLSX)

S1 Fig Conceptual bifactor model. (TIF)

S2 Fig Structural model after removing RRS 016, NEO1, NEO31. (TIF)
