# Supplementary figures and images for: Exploring the optimal factor structure of mind-wandering: Associations with neuroticism
Source: PLoS One. 2024 Dec 11;19(12):e0311733. doi: 10.1371/journal.pone.0311733 (PMC11633954; doi:10.1371/journal.pone.0311733)

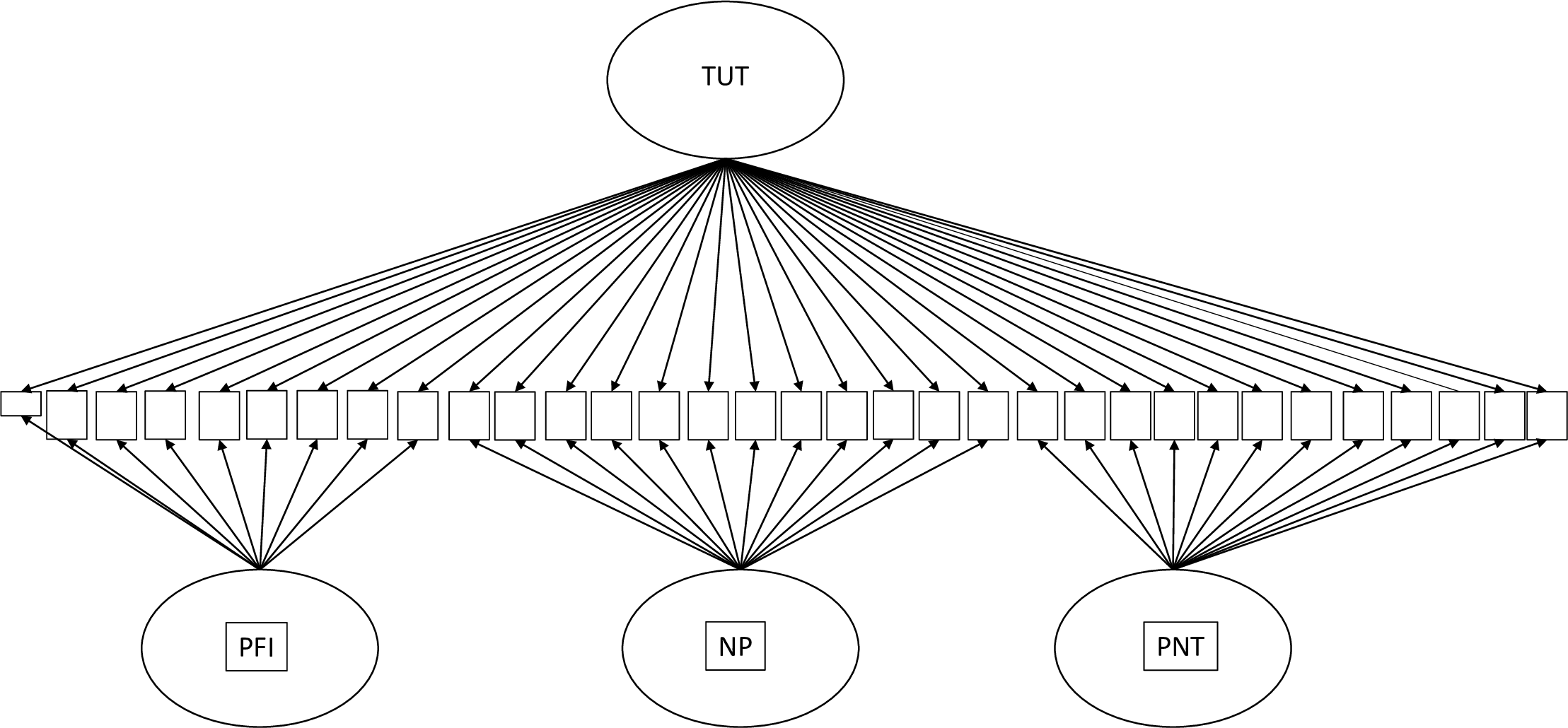

Supplement: S1 Fig — (TIF) [file pone.0311733.s015.tif]

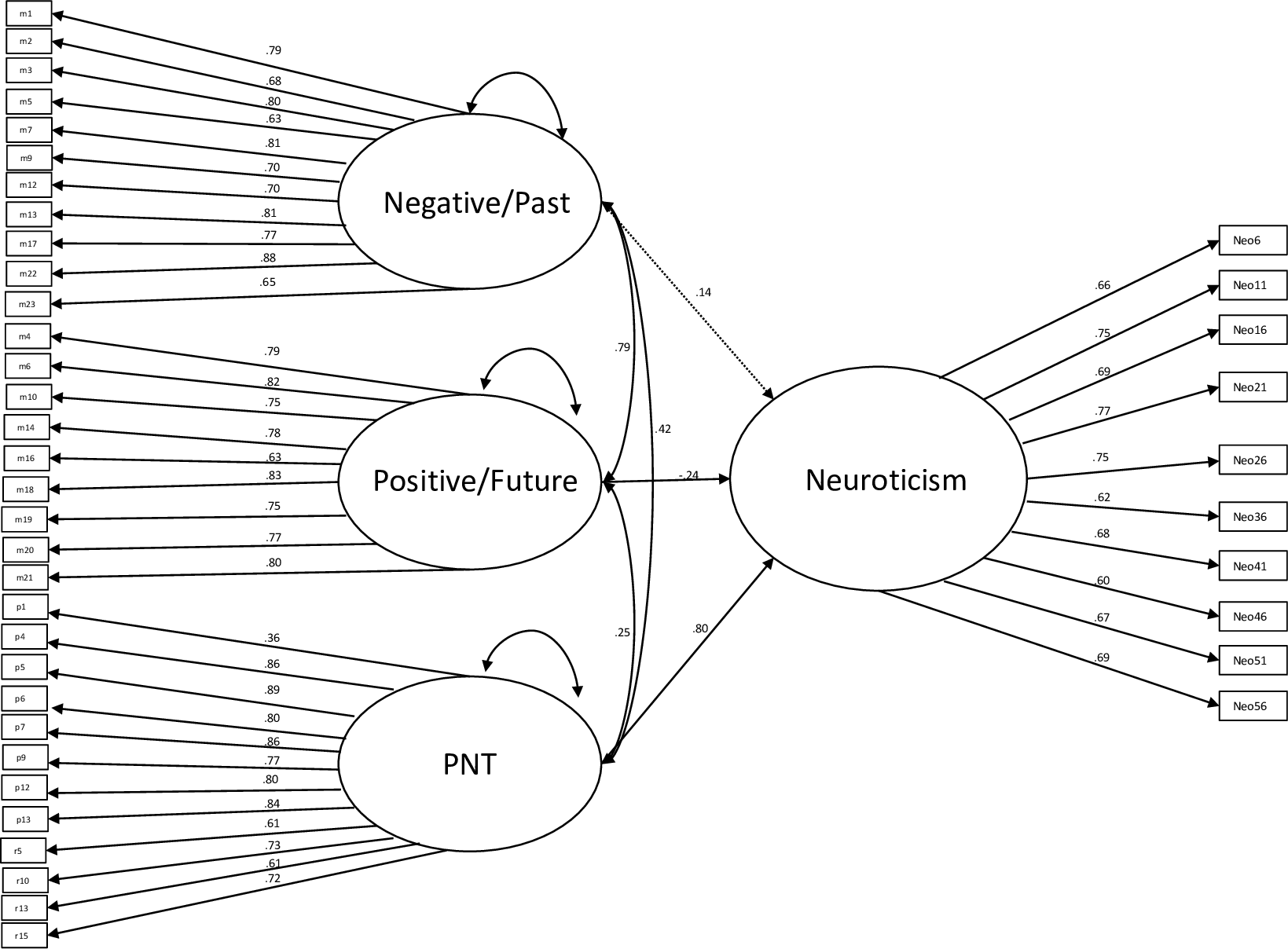

Supplement: S2 Fig — (TIF) [file pone.0311733.s016.tif]
